# Agent-Oriented Privacy-Based Information Brokering Architecture for Healthcare Environments

**DOI:** 10.1155/2009/101382

**Published:** 2009-03-23

**Authors:** Abdulmutalib Masaud-Wahaishi, Hamada Ghenniwa

**Affiliations:** ^1^College of Information Technology, United Arab Emirates University, P.O. Box 15551, Al-Ain, United Arab Emirates; ^2^Department of Electrical & Computer Engineering, The University of Western Ontario, 1201 Western Road, London, ON, Canada N6G 1H1

## Abstract

Healthcare industry is facing a major reform at all levels—locally, regionally, nationally, and internationally. Healthcare services and systems become very complex and comprise of a vast number of components (software systems, doctors, patients, etc.) that are characterized by shared, distributed and heterogeneous information sources with varieties of clinical and other settings. The challenge now faced with decision making, and management of care is to operate effectively in order to meet the information needs of healthcare personnel. Currently, researchers, developers, and systems engineers are working toward achieving better efficiency and quality of service in various sectors of healthcare, such as hospital management, patient care, and treatment. This paper presents a novel information brokering architecture that supports privacy-based information gathering in healthcare. Architecturally, the brokering is viewed as a layer of services where a brokering service is modeled as an agent with a specific architecture and interaction protocol that are appropriate to serve various requests. Within the context of brokering, we model privacy in terms of the entities ability to hide or reveal information related to its identities, requests, and/or capabilities. A prototype of the proposed architecture has been implemented to support information-gathering capabilities in healthcare environments using FIPA-complaint platform JADE.

## 1. Introduction

Healthcare systems are characterized by shared and distributed decision making and management of care. The distributed nature of the knowledge among different healthcare locations implies that a request may not be completely satisfied at a specific location or that one or more healthcare location may contain information similar to, though not exactly the same as, that required by the request.

Many initiatives and programs have been established to promote the development of less costly and more effective healthcare networks and systems at national and international scales. The objectives of these healthcare networks are to improve diagnosis through online access to medical specialists, online reservation of analysis and hospital services by practitioners extended on wide global scale, transplant matching, and so forth. A complete electronic medical patient-case file, which might be shared between specialists and can be interchanged between hospitals and with general practitioners (GPs), will be crucial in diagnosing diseases correctly, avoiding duplicative risky and expensive tests, and developing effective treatment plans.

However, medical patient-case files may contain some sensitive information about critical and vital topics such as abortions, emotional and psychiatric care, sexual behaviors, sexually transmitted diseases, HIV status, and genetic predisposition diseases. Privacy and the confidentiality of medical records have to be especially safeguarded. Without broad trust in medical privacy, patients may avoid crucial healthcare provision.

Healthcare professionals and care providers prefer to have the ability of controlling the collection, retention, and distribution of information about themselves. On the other hand, healthcare service providers need to effectively manage and prevent any abuse of the information or service they provide in addition to the ability of protecting their identities. An important feature of the various healthcare sectors is that they share similar problems and are faced with challenges that can be characterized as follows.

(i) In open-distributed healthcare environments, it is no longer practical to expect healthcare clinicians, staff, care providers, and patients to determine and keep track of the information and services relevant to his/her requests and demands. For example, a patient will be ubiquitously able to access his/her medical record from anywhere at any time or may request medical services offered by available healthcare centers in a particular city without being aware of the distributed sources and irrespective of their locations. In addition, an application should be able to manage distributed data in a unified fashion. This involves several tasks, such as maintaining consistency and data integrity among distributed data sources, and auditing access.

(ii) The distributed nature of the knowledge among multiple healthcare locations may require collaboration for information gathering. For example, each unit in a hospital keeps its own information about patients' records.

(iii) The solution of specific medical problem includes complex activities and requires collaborative effort of different individuals who posses distinct roles and skills. For example, the provision of care to hospitalized patients involves various procedures and requires the coordinated interaction amongst various staff and medical members. It is essential that all the involved medical staff and professionals must coordinate their activities in a manner that will guarantee the best appropriate treatment that can be offered to the patient.

(iv) A recent survey shows that 67% of the American national respondents are concerned about the privacy of their personal medical records, 52% fear that their health insurance information might be used by employers to limit job opportunities, while only 30% are willing to share their personal health information with health professionals not directly involved in their case. As few as 27% are willing to share their medical records with drug companies [1].

To explore such issues, distributed healthcare systems need to have an access to a service that can enable collaboration between different healthcare service requesters and providers. Brokering facilitates achieving better coordination among various healthcare service requesters and providers, and permits healthcare personnel to get access to different services managed by various providers without having to be aware of the location, identities, access mechanisms, or the contents of these services.

The proactive health systems have the potential to improve healthcare access and management which significantly lower the associated incurred costs through efficiently controlled information flow between various physicians, patients, and medical personnel, yet threaten to facilitate data sharing beyond any privacy concerns. The high degree of collaborative work needed in healthcare environments implies that developers and researchers should think of other venues that can manage and automate this collaboration efficiently.

However, privacy concerns over inappropriate use of the information make it hard to successfully exploit and achieve the gains from sharing such information. This dilemma restricts the willingness of individuals and personnel to disseminate or publicize information that might lead to adverse outcomes. This paper presents an agent privacy-based information brokering architecture that supports ad hoc system configurations emphasizing the strategies for achieving privacy in healthcare environments. Within the context of brokering, we view privacy as “*the ability of entities to decide upon revealing or hiding information related to their identities, requests and capabilities in open distributed environments.*”

## 2. Related Work

Privacy concerns are key barriers to the growth of health-based systems. Legislation to protect personal medical information was proposed and put in effect to help building a mutual confidence between various participants in the healthcare domain.

Privacy-based brokering protocols were proposed in many application domain such as E-auctions [2], data mining [3], and E-commerce. Different techniques were used to enable collaboration among heterogeneous cooperative agents in distributed systems including brokering via middle agents. These middle agents differ from the role they play within the agent community [4–6]. The work in [7] has proposed an agent-based mediation approach, in which privacy has been treated as a base for classifying the various mediation architectures only for the initial state of the system. In another approach, agents capabilities and preferences are assumed to be common knowledge, which might violate the privacy requirements of the involved participants [8]. Other approaches such as in [9–11] have proposed frameworks to facilitate coordination between web services by providing semantic-based discovery and mediation services that utilize semantic description languages such as OWL-S [12] and RDF [13]. Another recent approach distinguishes a resource brokering architecture that manages and schedules different tasks on various distributed resources on the large-scale grid [14]. However, none of the above-mentioned approaches has treated privacy as an architectural element that facilitates the integration of various distributed systems of an enterprise.

Several approaches were proposed for integration of distributed information sources in healthcare [15]. In one approach [16], the focus was on providing management assistance to different teams across several hospitals by coordinating their access to distributed information. The brokering architecture is centralized around a mediator agent, which allocates the appropriate medical team to an available operating theatre in which the transplant operation may be performed. Other approach attempts to provide agent-based medical appointments scheduling [17, 18], in these approaches the architecture provides matchmaking mechanisms for the selection of appropriate recipient candidates whenever organs become available through a matchmaking agent that accesses a domain-specific ontology.

Other approaches proposed the use of privacy policies along with physical access means (such as smartcards), in which the access of private information is granted through the presence of another trusted authority that mediate between information requesters and information providers [19, 20]. A European IST project [21], TelemediaCare, Lincoln, UK, developed an agent-based framework to support patient-focused distant care and assistance, in the architecture composes two different types of agents, namely, stationary “static” and mobile agents. Web service-based tools were developed to enable patients to remotely schedule appointments, doctor visits, and to access medical data [22].

Different approaches had been suggested to protect the location privacy in open-distributed systems [23]. Location privacy is a particular type of information privacy that can be defined as “the ability to prevent other parties from learning one's current or past location”. These approaches range from anonymity, pseudonymity, to cryptographic techniques. Some approaches focus on using anonymity by unlinking user personal information from their identity. One available tool is called anonymizer [24]. The service protects the Internet protocol (IP) address or the identity of the user who views web pages or submits information (including personal preferences) to a remote site. The solution uses anonymous proxies (gateways to the Internet) to route user's Internet traffic through the tool. However, this technique requires a trusted third party because the anonymizer servers (or the user's Internet service provider, ISP) can certainly identify the user. Other tools try not to rely on a trusted third party to achieve complete anonymity of the user's identity on the Internet, such as Crowds [25], Onion routing [26], and MIX networks [27].

Various programs and initiatives have proposed a set of guidelines for secure collection, transmission, and storage of patients' data. Some of these programs include the Initiative for Privacy Standardization in Europe (IPSE) and the Health Insurance Portability and Accountability Act (HIPAA) [28, 29]. Yet, these guidelines need the adoption of new technology for healthcare requester/provider interaction.

## 3. Brokering Requirements for Distributed Healthcare Systems

Brokering enables collaboration between different service requesters and providers, and allows the dynamic interpretation of requests for the determination of relevant service providers. For service providers, the brokering services permit dynamic creation of services' repositories after suitable assembly of service advertisements available from the various providers, or other additional activities. The major functional requirements of a brokering service include the following.

(i) *Provision of registration services*: the registration and naming service allows building up a knowledge base of the environment that can be utilized to facilitate locating and identifying the relevant existing service sources and their contents for serving a specific request. It is crucial to be able to identify the subset of relevant information at a source, and to combine partially relevant information across different sources; this requires the process of identification and retrieval of a subset of required service at any source. It is clear that in such environment, different sources would provide relevant information to a different extent. The most obvious choice of the source from which information will be retrieved is the one which returns most (or all) of the relevant request. In that case, the user will have to keep track of which source has the most relevant information.

(ii) *The acceptance of providers' service descriptions*: to enable the dynamic discovery of services, a mechanism is required to describe the capability aspects of services, such as the functional description of a service, the conditions and the constraints of the service, and the nature of the results.

(iii) *Receiving services' requests*: to enable requesters to define and describe the required parameters that are needed to represent a request.

(iv) *Interaction*: brokers may engage (on behalf of requesters) in the process of negotiation with various service providers to serve a request. The interaction requires a set of agreed messages, rules for actions based upon reception of various messages.

(v) *Communication*: the communication capability allows the entities to exchange messages with the other elements of the environment, including users, agents, and objects. In order to perform their tasks, these entities need to depend heavily on expressive communication with others not only to perform requests, but also to propagate their capabilities, advertise their own services, and explicitly delegate tasks or requests for assistance.

## 4. The Brokering Layer: Privacy-Based Agent-Orinted Architecture

Developing the brokering services comprises the automation of privacy to enhance the overall security of the system and accordingly entities should be able to define the desired degree of privacy. In fact, the brokering service permits entities to participate in the environment with different roles, and hence be capable of automating their privacy concerns and select a particular privacy. The challenge here is how to architect a service that could provide means and mechanisms by which entities would be able to interact with each other and determine any privacy degree that suits a particular situation. Such interaction is characterized by the nondeterministic aspect in addition to the dynamic nature of the environment, where these entities exist and operate for which they require to be able to change configurations to participate in different roles. These requirements could not be accomplished using traditional ways of manually configuring software.

We strongly believe that agent orientation is an appropriate design paradigm for providing coordination services and mechanisms in such settings. Indeed, such a paradigm is essential to modeling open, distributed, and heterogeneous environments in which an agent should be able to operate as a part of a community of cooperatively distributed systems environments, including human users. A key aspect of agent orientation is the ability to design artifacts that are able to perceive, reason, interact, and act in a coordinated fashion. Here, we view agent orientation as a metaphorical conceptualization tool at a high level of abstraction (knowledge level) that captures supports and implements features that are useful for distributed computation in open environments. These features include cooperation, coordination, interaction, as well as intelligence, adaptability, economic and logical rationalities. We define an agent as an individual collection of primitive components that provide a focused and cohesive set of capabilities. We focus on the notion of agenthood as a metaphorical conceptualization tool at a high level of abstraction (knowledge level) that captures supports and implements features that are useful for distributed computation in open environments.

Architecturally, the brokering service is viewed as a layer of services and is modeled as an agent with a specific architecture and interaction protocol that are appropriate to carry the required privacy degree. The challenge in this context is how to architect the brokering layer with the appropriate set of services that enable cooperation across the different degrees of privacy. The interaction protocols represent both the message communication and the corresponding constraints on the content of messages. They describe the sequence of messages among agents, and illustrate various protocols that satisfy a desired privacy requirement. The focus for designing these patterns is to provide a mechanism to reduce the costs and risks that might be a result of violating privacy requirements. The patterns provide mechanisms allowing users (human/agents) to adjust the privacy attributes, and allowing these users to achieve and accomplish their tasks in addition to protecting their desired privacy attributes.

The agent interaction requires a set of agreed messages, rules and assumption of communication channels. These rules and constraints can be abstracted as agents' patterns that define various protocols for every possible privacy requirement. Using these protocols, agents would be able to protect the privacy aspects of the most concern. From the privacy standpoint, the brokering services are categorized into different roles that are classified according to the participants' (providers and requesters) desired degree of privacy. These degrees of privacy control the proper interaction patterns and will vary from a specific scenario to another. The brokering layer takes in consideration the protection of any privacy desires required by requesters, providers, or both.

Here, we define the degree of privacy in terms of three attributes: the entity identity, capability, and goals. Therefore, an agent can categorize its role under several privacy degrees. Formally, an agent can be represented as a 2-tuple *Ag* ≡ 〈(*RA* : *Id*, *G*); (*PA* : *Id*, *Cap*)〉, where *RA* and *PA* refer to the agent role as requester and provider while *Id*, *G*, and *Cap*, respectively, refer to the agent identity, goals, and capabilities, which might have a null value. For example, an agent might participate with a privacy degree that enables the hiding of its identity as a requester by setting the value of *Id* to null. Tables 1, 2 summarize the different scenarios and roles that might be played by the brokering layer categorized by the possible privacy concern of the requester (*RA*) and provider (*PA*) agents.

The layer permits various entities to participate in the environment with different roles, and hence be capable of automating their privacy concerns and select a particular degree. Each layer role is represented as a special broker with a specific architecture and interaction protocol that is appropriate to serve requests from various participants while maintaining the required privacy degree. An agent role is an abstract description of an entity with the specified functionalities. The brokering layer has the ability to interact, solicit help, and delegate services' requests from other available brokering agents who support different privacy degrees.

Responsibilities are separated and defined according to the roles played and the required degree of privacy. Within the layer two sets of brokering agents are available to service requesters and providers. The first set handles interactions with requesters according to the desired privacy degree that is appropriate to their preferences while the other set supports privacy degrees required by service providers.

**Figure 1:**
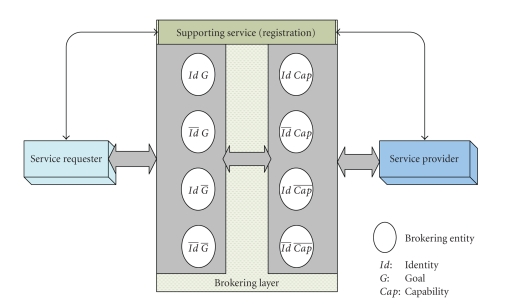
Logical view of the brokering service.

Figure 1 shows a logical view of the brokering services and the relevant entities that are involved in any brokering scenario. Every brokering pattern is accomplished by the composition of the requester role, brokering agents, and the provider role, in which the interaction scenarios are produced automatically. A complete brokering session is divided into several stages, starting from requester-to-brokering layer interaction, brokering layer intra-interaction, and broker layer-to-provider interaction. Note that in the figure a negation on a specific privacy attribute variable exemplifies that the corresponding privacy attribute is hidden from the environment.

## 5. The Brokering Protocols: Privacy-Based Interaction Patterns

The brokering protocols describe a cooperative multibrokering system, which provides the solution for interaction among participants in a dynamic and heterogeneous environment of service providers and requesters. Each brokering entity performs basic brokering functionality, such as service discovery, dynamic service composition, and knowledge sharing with the community according to a required privacy degree. A brokering entity within the layer is called a broker hereafter.

Brokers within the layer might represent a set of services in which providers can advertise their service capability. The brokering protocols regulate and govern service knowledge discovery and sharing of acquired knowledge by defining interaction patterns that are composed of a set of messages that can be exchanged by other brokers within the layer or other registered entities that might benefit of the functionalities supported by the overall brokering service. The architecture permits the brokering agents to have various combinations with other brokering entities which support different privacy degrees. The following section describes the different interaction patterns supported by the brokering layer for entities that might play either a requester or a provider role.

### 5.1. The Requester-Brokering Layer Interaction

#### 5.1.1. Requesters Revealing Identities and Goals

The broker protects the privacy of healthcare personnel, patients, or staff. It assists service requesters to achieve their goals without exposing their identities to the environment. For example, information about the number of patients who have Hepatitis B in a specific city and wanted by a doctor can be assessed by the broker agent without revealing, neither the doctor nor the patients identities. However, agents playing the role of requesters and wanting to benefit from such a service are required to reveal their identities and goals to the relevant broker within the layer. Note that each privacy degree is described in terms of two main interactions: an interaction amongst the various brokers within the brokering layer (intra-interaction) and the interaction between the domain (i.e., a requester or a provider) with the relevant broker that supports a particular privacy degree (inter-interaction).

**Intra-Interaction**

**Figure 2:**
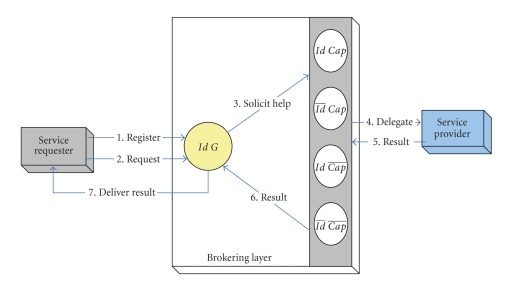
Interaction pattern for requesters revealing privacy attributes.

As shown in Figure 2, the broker might extend the pattern to include interaction with various brokers associated with supporting other privacy degrees of service providers, consequently the broker solicit help and forward request to all available provider-related brokers within the layer incorporating various interaction compositions. Note that for every potential composition, the provider-related brokers receive only a notification of a service request, and accordingly carry on its own interaction pattern to satisfy that request without exaggerating, overstressing, or overemphasizing any incurred rights or privileges (e.g., cost).

**Inter-Interaction**

The typical interaction pattern for this particular privacy degree comprises that the layer engages in performing the following: (1) accepting and interpreting service requests from pertinent requesters; (2) identifying and contacting a set of available providers, forwarding service requests, and controlling appropriate transactions to fulfill any required service request. These transactions should adhere to agreed appropriate interaction mechanism (e.g., auction, negotiation, etc.); (3) receives result of a service request and delivers it back to the relevant requester.

#### 5.1.2. Requesters Hiding Identities

**Figure 3:**
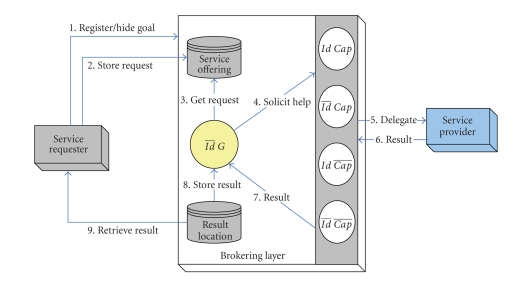
Interaction pattern for requesters hiding identity.

Requesters such as patients with fatal diseases may wish to access services or seek further assistance without revealing their identities. The brokering service dynamically identifies relevant service providers, and acts on behalf of those requesters to fulfill their goal(s). As shown in Figure 3, requesters will be responsible of checking the availability of the service result, which implies that requesters should be aware of a designated result location. The interaction imposes a significant effort on the performance and efficiency. System performance is clearly dependent on number of parameters, including the number of providers willing to carry out the request and the time needed by each provider to fulfill that request.

**Intra-Interaction**

As described in the previous case, the broker might extend its pattern to include an interaction composition with various brokers associated with supporting other privacy degrees for service providers. Upon receiving a service result, the broker stores the result in a dedicated repository (result repository) to be retrieved by the relevant requester.

**Inter-Interaction**

Requesters may wish to access services or seek further assistance without revealing their identities. The interaction pattern for this particular privacy degree is as follows: (1) requesters are required to store services requests in a predefined service repository along with preferred parameters. (2) As shown in Figure 3, requesters are responsible of checking the availability of the service result and hence retrieve it; this implies that requesters are able to link a service result to their own requests.

#### 5.1.3. Requesters Hiding Goals

There might be certain situations where requesters prefer to hide their goals from the environment; the layer functionality entails the forwarding of every advertised service out to every registered requester with unknown preferences or interests. For example, clinician might benefit from variety of service advertisements regarding new medications, tools, medical equipments, and health-related notifications. The brokering service permits these clinicians to check a service repository for further information or to browse other service offerings that have been previously posted and accordingly determine an appropriate and interested service.

**Intra-Interaction**

Provider-related brokers representing providers with known capabilities will have the possibility to advertise existing service offerings to the broker which in turn promotes forwarding every received advertisement to the relevant requester. It is to be noted that whenever a requester decides on a particular service offering, the inter-interaction is not restricted only to contacting those who had offered such services, but might extend to all available provider-related brokers supporting other privacy degrees. For example, the same advertised service offering might be achieved by other providers in the environment who had the interest of hiding their own capabilities.

**Inter-Interaction**

**Figure 4:**
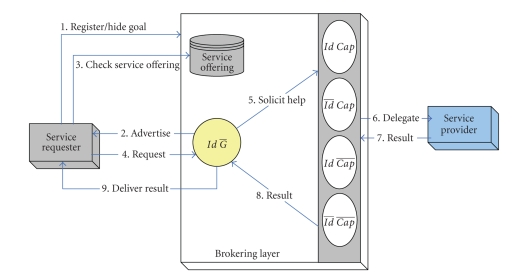
Interaction pattern for requesters hiding goals.

They broker permits healthcare requesters to check a service repository for further information or to browse other service offerings that have been previously posted and accordingly determine an appropriate and interested service as shown in Figure 4. Once a requester selects a particular service advertisement and forwards that request to the broker, then it is the broker responsibility to determine the most suitable service provider that fulfills that request. Upon achieving the requester goal, the broker delivers back the service result to the requester. In an open environment, where many different services providers are in continual increase and with a competitive manner to sell their services, requesters would be flooded by a variety of service advertisements and notifications. Requesters have to determine whether the service advertised to them is of an interest or not. Clearly, this process implies that a significant time is required to assess every single-service notification. The broker sends the notifications along with any related parameters required for providing the service (such as name of the service, cost, and location).

#### 5.1.4. Requesters Hiding Identities and Goals

**Figure 5:**
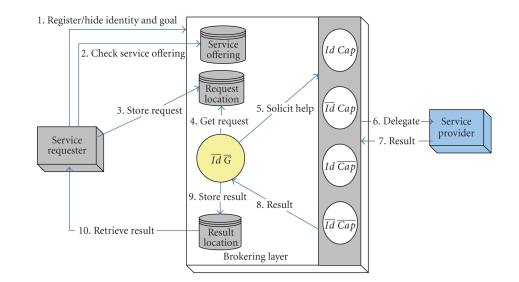
Interaction pattern for requesters hiding privacy attributes.

Requesters would have the possibility to hide their identities and goals from the entire environment; as shown in Figure 5, they have the option either to post their want ads to the layer service repository directly, or might check for any services that would be of an interest. For example, patients with narcotic-related problems (such as drug or alcohol addiction) can seek services that provide information about rehabilitation centers, specialized psychiatrists, or programs that will help overcoming a particular critical situation without revealing either their identities nor the desired information.

**Inter-Interaction**

Requesters will have the option to either post their want ads to a service repository directly, or might check for any service offerings that would be of an interest. In both cases, requesters will be permitted to store their service requests and retrieve services results. The broker identifies and interprets the required requests, and accordingly will determine the applicable provider which is capable of achieving and fulfilling the requester goal. Note that, for this degree of privacy, it is the requester responsibility to check for the availability of the service result, and hence retrieve it.

### 5.2. The Provider-Brokering Layer Interaction

#### 5.2.1. Providers Revealing Identities and Capabilities

Providers with this degree of privacy will have the ability to register their presence along with the capability of the service they offer. Although providers with this privacy degree are required to reveal their privacy attributes to the relevant broker, the protocol will suppress any other entity from knowing the provider attributes.

**Intra-Interaction**

The interaction between the broker and other requester-related brokers is accomplished through sending and receiving messages related to service proposals, service offerings, and services results.

**Inter-Interaction**

**Figure 6:**
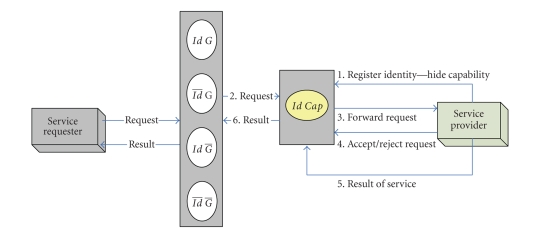
Interaction pattern for providers revealing privacy attributes.

As shown in Figure 6, a service provider registers itself with the broking service, along with the description of its service capabilities which is stored as an advertisement in a repository maintained by the broker and contains all available service descriptions. Assigning requests to providers with known capabilities and identities can be based on either broadcasting or focusing, however, the interaction is neither restricted to specific service providers nor committed to a fixed number of them. This ability is particularly useful in which a brokering agent acts in a dynamic environment in which entities may continually enter and leave the society unpredictably. For every received service request, the broker matches the most applicable providers that are appropriate to fulfill that request, and thus maintains a pertinent queue that contains the capable providers along with their identities.

#### 5.2.2. Providers Hiding Identities

Healthcare providers can have the option to hide their identities from the environment and advertise their service offerings to the relevant brokering agent. Protection for the core identity prevents service abuses that impact availability of service and hence improving the ability to consistently deliver reliable access. Since the service capabilities are known to the broker, service requests that are believed to be fulfilled by such providers will be posted to a dedicated repository for which providers will have the possibility to browse such requests and select whichever of an interest.

**Intra-Interaction**

The broker interacts with other entities in the layer to engage in receiving and sending messages related to service requests and offerings. The broker task includes (1) receiving service requests; (2) determining whether these requests are within the provider capabilities; (3) storing service requests to be browsed by authorized registered providers (providers hiding identities); (4) retrieving and delivering back service result. A broker supporting this privacy case will have the ability to advertise registered provider capabilities, and hence engage in various interaction patterns of available requester-related brokers.

**Inter-Interaction**

**Figure 7:**
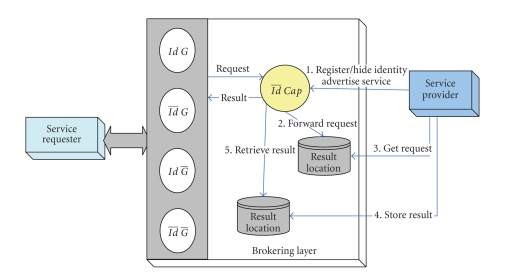
Interaction pattern for providers hiding identity.

A provider can participate in any interaction mechanism and may respond to call-for-proposal requests by proposing service offerings that are stored in a queue-structured repository. Upon assigning and delegating a service request to a provider with this degree of privacy, it is the provider responsibility to store pertinent service result to be retrieved by the broker, and thus delivered to the proper destination as shown in Figure 7.

#### 5.2.3. Providers Hiding Capabilities

**Figure 8:**
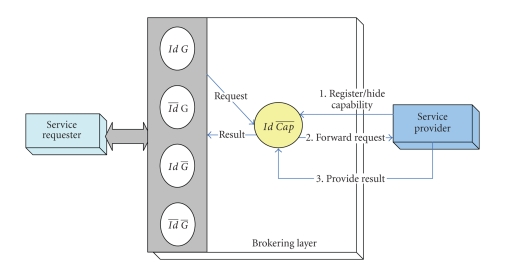
Interaction pattern for provider hiding capability.

The brokering services allow providers that do not wish to reveal their own capabilities to participate in fulfilling a service request. After receiving a request, the brokering interaction protocol exemplifies the forming out of requests to every registered provider with unknown capability. It is noteworthy that, for every advertised request, providers have to determine whether the request is within their capabilities and/or of an interest. Clearly, such an interaction implies that a considerable elapsed time will be spent on evaluating every single request. Therefore (under the assumption of an open dynamic environment), providers would be deluged by a variety of service requests, which significantly impact performance and efficiency. Figure 8 shows the interaction pattern.

**Intra-Interaction**

The broker interacts with other entities in the layer to engage in receiving and sending messages related to service requests and offerings. The broker task includes (1) receiving service requests from requester-related brokers; (2) receiving service proposals; (3) delivering back service result.

**Inter-Interaction**

After receiving a service request, the broker sends out requests in the form of broadcasting to every registered provider with unknown capabilities. Figure 8 shows the interaction pattern. Once a provider selects a particular service request, it forwards a service proposal to the broker who controls the remaining transaction according the appropriate negotiation mechanisms similar to what has been described in the former patterns.

#### 5.2.4. Providers Hiding Identities and Capabilities

**Figure 9:**
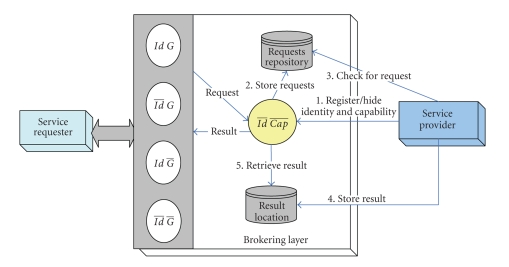
Interaction pattern for provider hiding privacy attributes.

Providers will have the ability to browse a special request repository and consequently determine the relevant requests that might be of an interest and within their capabilities. As shown in Figure 9, the broker-provider side agent responds back with the service result (a result location within the layer has to be identified to the provider upon registration within the brokering layer).

**Intra-Interaction**

The broker intra-interaction comprises the following: (1) receiving service requests from requester-related brokers; (2) storing service requests; (3) accessing and evaluating service proposals; (4) retrieving and delivering back service result.

**Inter-Interaction**

In this protocol, the brokering functionality is mainly seen as a directory service, in which the broker maintains a repository of service requests along with any required preferences. Providers will have the ability to browse this repository to determine applicable relevant requests that might be fulfilled. As shown in Figure 9, providers with this degree of privacy have to take in consideration linking the result of the service to the request.

## 6. Design and Implementation

### 6.1. Modelling Healthcare-Distributed Systems

It is clear that the development of coordination solutions in open and distributed healthcare environments requires a new design paradigm, improved integration architectures and services. A cooperative distributed systems (CDSs) approach is an ideal and appropriate design paradigm which allows the various healthcare entities to exercise some degree of authority in sharing their information and capabilities.

The architecture must describe the organization and the interconnection among the software entities. In this architecture, the environment can be envisioned as a cooperative distributed system (CDS) comprised of a collection of economically motivated software agents that interact competitively or cooperatively, find and process information, and disseminate it to humans and other agents. It also enables common services that facilitate the coordination and the cooperation activities amongst various domain entities and support ad hoc and automated configurations.

In our proposed model, a CDS is conceptualized as a dynamic community of agent and nonagent entities that contribute with different services. Based on the above view, an agent might play different roles and be able to coordinate cooperatively or competitively with other agents, including humans. Therefore, healthcare CDS entities are mapped as follows.

(i) *Service requester*: is a domain specific entity that can interact with the environment and request services.

(ii) *Service provider*: a domain entity that provide application-specific services.

(iii) *Brokering entity*: is an agent that provides common coordination services, and facilities for the generic cooperative distributed systems environment.

### 6.2. The Coordinated Intelligent Rational Agent (CIR-Agent) Model

The representative agents of domain and brokering entities within the context of healthcare-based CDS are built on the foundation of CIR-agent architecture with focuses on utilizing the model to capture the participants' individual behavior toward achieving a desirable goal while maintaining a required privacy degree.

**Figure 10:**
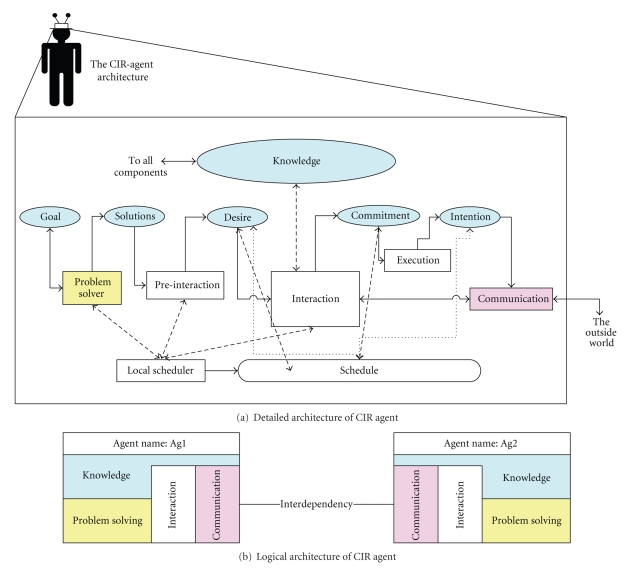
The CIR agent architecture.

The CIR-agent is an individual collection of primitive components that provide a focused and cohesive set of capabilities. The basic components include problem-solving, interaction, and communication components, as shown in Figure 10(b). A particular arrangement (or interconnection) of components is required to constitute an agent. This arrangement reflects the pattern of the agent mental state as related to its reasoning about achieving a goal. However, no specific assumptions need to be made on the detailed design of the agent components. Therefore, the internal structure of the components can be designed and implemented using object oriented or another technology, provided that the developer conceptualizes the specified architecture of the agent as described in Figure 10.

Basically, each agent consists of knowledge and capability components. Each of which is tailored according to the agent specific role. The agent knowledge contains information about the environment and the expected world. The knowledge includes the agent self-model, other agents' model, goals that need to be satisfied, possible solutions generated to satisfy each goal, and the local history of the world that consists of all possible local views for an agent at any given time. The agent knowledge also includes the agent desires, commitments, and intentions toward achieving each goal. The capability package includes the reasoning component; the domain actions component which contains the possible set of domain actions that when executed, the state of the world will be changed; the communication component where the agent sends and receives messages to and from other agents and the outside world.

The problem solver component represents the particular role of the agent and provides the agent with the capability of reasoning about its knowledge to generate appropriate solutions directed to satisfy its goal. During the interaction processes, the agents engage with each other while resolving problems that are related to different types of interdependencies. The coordination mechanisms are meant to reduce and resolve the problems associated with interdependencies. Interdependencies are goal-relevant interrelationships between actions performed by various agents.

As argued in [30], the agent interaction module identifies the type of interdependencies that may exist in a particular domain. Consequently, agents select an appropriate interaction device that is suitable to resolve a particular interdependency. (Interaction device is an agent component by which it interacts with the other elements of the environment through a communication device. A device is a piece or a component with software characteristics that is designed to service a special purpose or perform a special function). These devices are categorized as follows.

- Contract based includes the assignment device.
- Negotiation based includes resource scheduling, conflict resolution, synchronization, and redundancy avoidance devices.

**Figure 11:**
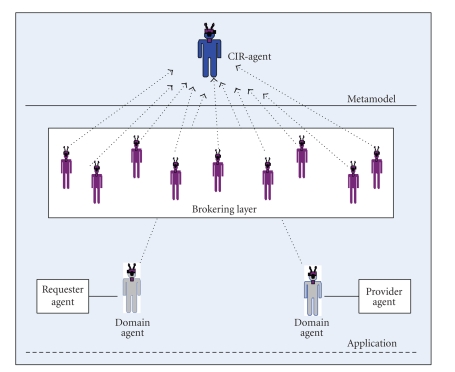
The overall system model.

Within the context of brokering, the interdependency problem is classified as capability interdependency, and the interaction device is the “assignment”. The basic characteristics of the assignment device are problem specifications, evaluation parameters, and the subprocesses. The problem specifications might include, for example, the request, the desired satisfying time, and the expiration time. A collection of basic components comprises the structure of the agent model and represents its capabilities. The agents architectures are based on the CIR-agent model as shown in Figure 11. A brokering session mainly recognizes two types of agents, namely, domain agent (requester or provider) and brokering agent (ReqBroker or ProvBroker). The architecture of each agent type is described in detail below.

#### 6.2.1. The Domain Agent: Service Providers and Requesters

**Figure 12:**
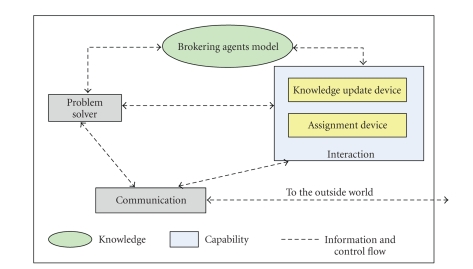
The domain agent architecture.

Service providers and requesters are modeled as domain agents as shown in Figure 12. The requester agent can participate with various privacy degrees and request services from the brokering layer. A requester delegates the service request(s) to the relevant brokering agent according to the interaction protocol of the selected privacy degree. The domain agent possesses knowledge and capability. The knowledge includes the model of the brokering agents in terms of the supported privacy degree, self-model, and the local history. The capability is categorized into three components: reasoning that includes problem-solving and coordination, communication, and a set of domain actions.

A domain agent playing the role of a service provider can select the appropriate privacy degree, and thus participate in providing the capability that meets the needs of another domain entity. The problem solver of the domain agent hiding any of the privacy attributes encompasses the accessing of different storage repositories. For example, the problem solver of a requester includes functionalities related to formulating service requests, checks for available service offerings, and accesses various storage repositories to store requests or to retrieve service results. On the other hand, the problem solver of a provider hiding its identity and capability attributes consists of modules related to accessing storage repositories to check for stored service requests that might be fulfilled and hence participating in storing service proposals and service results.

The coordination component of a requester comprises the interaction device which entails soliciting service from the relevant ReqBroker agent. The interaction device of the provider agent manages the coordination activities which involve proposing services to specific CFP messages and engage in bidding processes.

#### 6.2.2. The Brokering Agents: ReqBrokers and ProvBrokers

**Figure 13:**
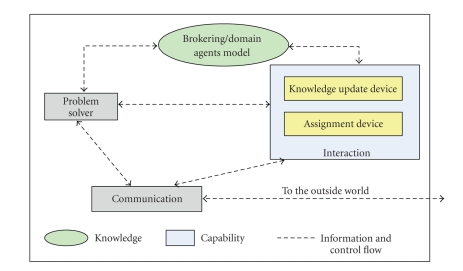
The brokering agent architecture.

A brokering agent is composed of two components, namely, the knowledge and capability. The knowledge component contains the information in the agent memory about the environment and the expected world. As shown in Figure 13, this includes the agent self-model, models of the domain agents in terms of their roles (requester/provider) and/or capabilities, and the local history of the world. The knowledge includes all possible local views for an agent at any given time (such as the knowledge of physical repositories, available services requests, services offerings, and service results).

#### 6.2.3. Implementation Example: Agent-Oriented Privacy Brokering for Healthcare CDS

In this section, we show an example of our proposed model applied to healthcare environments to support information-gathering capabilities. We describe the implementation of one pattern associated with an information requester hiding identities and goals and with three information providers; one is revealing privacy attributes, the second hiding its identity, while third is hiding its own privacy attributes (identities and capabilities). The broker agent (called ReqBroker henceforth) protects the privacy of requesters, understands the preferences, routes requests, and replies appropriately. All the inter-interactions utilize the FIPA Contract Net Protocol [13] as a negotiation mechanism. Consider an online three information providers, E-VirtualMedInfo Inc., E-VirtualDiagnosis Inc., and FutureDocAssistants Inc. (names are fictitious), each is represented by an agent.

The three providers offer medical-related information, healthcare guidelines, and clinical diagnosis procedures that can be supplied to various medical students, clinicians, staff, doctors, and physicians in various formats (online delivery, hard copies, or access to online medical repositories). All the three companies decided to register and subscribe to the brokering service and make use of the various privacy degrees. *E-VirtualMedInfo* registered with the brokering service while revealing it privacy attributes, *E-VirtualDiagnosis* comprises diagnosis capabilities jointly derived retired medical doctors and had selected hiding its identity, whereas *FutureDocAssistants*, a company that can also provide various online samples of medical exams and virtual evaluation assessments for different medical specialties, decided to hide both the identity and the capabilities. Upon registration, a dedicated brokering agent (ProvBroker) will be assigned to each company.

Alice, a four-year medical student, is conducting a research on the most top fatal diseases in Canada, the mortality and death rates of each and the possible diagnosis and prevention procedures that would help a trainee-student in examining and diagnosing patients with such diseases. Deciding to hide her own identity, Alice anonymously requests this information by posting the required information in special repository dedicated to such privacy degree.

After storing the request, Alice's assigned broker (ReqBroker) interacts with various ProvBrokers associated with supporting other privacy degrees of service providers (including the three mentioned companies) and consequently (acts as a manager) issues, and announces a call-for-proposals (CFPs) to those ProvBrokers (act as potential contractors) informing them of Alice's request specifications (note that Alice's identity is anonymous to each participant including its own supporting ReqBroker).

The announcement includes task abstraction, a brief description of the required information; bid specification, a description of the expected format of the information; expiration time, a specified time interval during which the required information is valid.

Each ProvBroker working on behalf of each company contacts the registered company agent and sends the request. Note that for the *FutureDocAssistants* company, the request is dispatched in special dedicate storing repository allowing its own agent to browse this repository and retrieve the request (if interested).

Every company (through its representing agent) determines the evaluation parameters (such as information quality, expiration time, and cost) and accordingly submits a bid along with the offer parameters (such as quality, cost, and availability). The *E-VirtualMedInfo* and *E-VirtualDiagnosis* agents will send the bids directly to their assigned ProvBrokers, while the *FutureDocAssistants* agent stores the bid in a repository that will be retrieved by the relevant ProvBroker.

Alice's dedicated ReqBroker receives those bids from every ProvBroker and carries on the evaluation process and accordingly determines the most bid (or bids) that fulfills Alice's request for all the interested, and sends back an acceptance-proposal message to the potential companies (winners) and a rejection-message to the bids that do not meet the evaluation parameters. After receiving the information that Alice was requesting, the ReqBroker stores it in a special repository for which she has a valid access to retrieve it without having to reveal her own identity or being exposed to the identity and the capabilities of the three companies which had participated in fulfilling her request.

A web-based prototype of the proposed system has been implemented using Jade [31], an FIPA [32] compliant, and Java Web Services Development Pack (JWSDP) platform to support and provide information-gathering capabilities to different participants in healthcare environments, where the accessibility of private information is a desirable feature to various categories of the healthcare personnel, patients, and clinicians.

**Figure 14:**
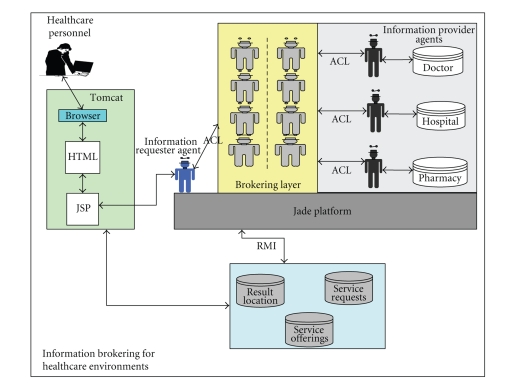
Privacy based brokering prototype for information gathering in healthcare.

The proposed architecture has been implemented using coordinated intelligent, rational agent (CIR-agent). As shown in Figure 14, three relational databases represent various medical data for three distributed locations, each being managed by a dedicated agent that can play both roles of an information requester as well as a provider.

Upon the required privacy degree and the role desired, the A Web interface is available for healthcare participants to select and register their desired privacy degree along with any information capability they might posses (medical data, patient diagnosis and treatment reports, pharmaceutical data reports, etc.). Based on the privacy degree required by the both the requester and information provider, a dedicated broker agent within the brokering layer will handle all the interaction required to fulfill an information request.

## 7. Discussion and Conclusion

Current advances in nowadays technologies coupled with the rapidly evolving healthcare paradigms allow us to foresee novel applications and services for improving the quality of daily life health activities. The increasing demand and dependency on information in healthcare organizations has brought the issues of privacy to every aspect of the healthcare environments. It is expected and with no doubt that medical data such as genome information, medical records, and other critical personal information must be respected and treated with a great concern. As awareness of the threats that organizations face becomes more well understood, the need for additional privacy specifications for open, distributed, and heterogeneous systems grows clear. Tremendous efforts have been devoted to privacy and security issues in distributed systems for the last few decades to find technological means of guaranteeing privacy by employing state-of-the-art encryption and anonymization technology. The proposed architecture provides feasible solution to privacy protection in open environments, and presents myriad of additional privacy and security opportunities without negative impact to the utilization of these services.

Architecturally, the proposed model is viewed as a layer of services, where different roles can be played by the various entities (requesters, brokers, and providers). While existing approaches provide traditional information brokering by incorporating agent-based solutions to make healthcare information more accessible to individuals, the proposed architecture classifies the brokering role into several subroles based on the attributes designated to describe the privacy-desired degree of both the information provider and the information requester. Each role is modeled as an agent with a specific architecture and interaction protocol that are appropriate to support a required privacy degree.

Within the layer, two sets of brokering entities are available to service requesters and providers. The first set handles interactions with requesters according to the desired privacy degree that is appropriate to their preferences, while the other set supports privacy degrees required by service providers. A brokering pattern is realized by the different roles played by the domain entities and their corresponding brokering agent. A complete brokering scenario is accomplished by performing different levels of interaction, namely, (1) requester-to-broker interaction, (2) broker-to-broker interaction, and (3) broker-to-provider interaction. Different combinations within the layer can take place to support the interbrokering interactions. The proposed layered architecture provides an appropriate separation of responsibilities, allowing developers and programmers to focus on modeling solutions and solving their particular application problems in a manner and semantics most suitable to the local perspective. Agent technology has been viewed as one of the key technologies for supporting information brokering in heterogeneous open environments. The use of agent technology provides high degree of decentralization of capabilities, which is the key to system scalability and extensibility.

Another important aspect of the model is that it treats the privacy as a design issue that has to be taken into consideration in developing healthcare information brokering systems. In healthcare environments, the proposed model provides feasible solution to the problem of information overload and privacy concerns. It enables transparent integration amongst different participants of healthcare CDS, and provides querying ability and coordination solutions that enhance the overall connectivity of distributed, autonomous, and possibly heterogeneous information sources (databases) of different healthcare sectors. It can efficiently govern different types of health-oriented information and critical medical data such as genetic, HIV, mental health, and pharmacy records from not distributed, disseminated, or abused. Based on the level and the amount of information that can be released, patients, clinicians, service providers, and medical staff members can securely translate their privacy policies to an applicable-related privacy case in the proposed model.

The proposed approach is innovative in the sense that it treats the privacy as a design issue for information brokering systems, and it supports ad hoc and automated configurations among distributed, possibly autonomous, and heterogeneous entities with various degrees of privacy requirements. The multilayer architecture minimizes the architecture complexity encountered in direct-interaction architectures (where interactions between agents often take more complex processes for encompassing series of message exchange and forming a single point of failure), and makes it less vulnerable to failures. The proposed layered architecture provides an appropriate separation of responsibilities, letting developers and programmers focus on solving their particular application problems in a manner and semantics most suitable to the local perspective.

## Figures and Tables

**Table 1:** The brokering layer interaction categorized by the privacy concern of service requesters.

	Privacy attributes		Interaction
Case	*G*	*Id*	
1	Revealed	Revealed	(i) Receive service request.
			(ii) Forward request to broker-provider side.
			(iii) Deliver result to requester.

2	Hidden	Revealed	(iv) Retrieve service request posted by a requester.
			(v) Forwards request to broker-provider side.
			(vi) Store result to be retrieved by requester.

3	Revealed	Hidden	(vii) Postservice request to service repository.
			(viii) Requester to search repository and request service.
			(ix) Retrieve a service request that was stored by a requester.
			(x) Forward request to available and capable providers.
			(xi) Store result to be retrieved by requester.

4	Hidden	Hidden	(xii) Requester to store service request.
			(xiii) Retrieve service request that was stored by a requester.
			(xiv) Forward request to available and capable providers.
			(xv) Store result to be retrieved by requester.

**Table 2:** The brokering layer interaction categorized by the privacy concern of service providers.

	Privacy attributes		Interaction
Case	*Id*	*Cap*	
1	Revealed	Revealed	(i) Search for capable provider.
			(ii) Forward request.
			(iii) Negotiate and assign a service request.
			(iv) Get service result and deliver result.

2	Hidden	Revealed	(v) Postservice request to service repository.
			(vi) Providers to access service repository.
			(vii) Providers to evaluate service parameters
			(viii) Store result.
			(ix) Brokering layer to retrieve and deliver result.

3	Revealed	Hidden	(x) Forward service request.
			(xi) Provider to evaluate request.
			(xii) Brokering layer to receive and deliver result back.

4	Hidden	Hidden	(xiii) Providers to access repository.
			(xiv) Provider to evaluate request.
			(xv) Provider to store service result.
			(xvi) Brokering layer to retrieve and deliver result back.
